# Kaiso Directs the Transcriptional Corepressor MTG16 to the Kaiso Binding Site in Target Promoters

**DOI:** 10.1371/journal.pone.0051205

**Published:** 2012-12-12

**Authors:** Caitlyn W. Barrett, J. Joshua Smith, Lauren C. Lu, Nicholas Markham, Kristy R. Stengel, Sarah P. Short, Baolin Zhang, Aubrey A. Hunt, Barbara M. Fingleton, Robert H. Carnahan, Michael E. Engel, Xi Chen, R. Daniel Beauchamp, Keith T. Wilson, Scott W. Hiebert, Albert B. Reynolds, Christopher S. Williams

**Affiliations:** 1 Division of Gastroenterology, Department of Medicine, Vanderbilt University Medical Center, Nashville, Tennessee, United States of America; 2 Department of Cancer Biology, Vanderbilt University Medical Center, Nashville, Tennessee, United States of America; 3 Department of Biochemistry, Vanderbilt University Medical Center, Nashville, Tennessee, United States of America; 4 School for Science and Math, Vanderbilt University Medical Center, Nashville, Tennessee, United States of America; 5 Section of Surgical Sciences, Department of Surgery, Division of Surgical Oncology, Vanderbilt University Medical Center, Nashville, Tennessee, United States of America; 6 Department of Biostatistics, Vanderbilt University Medical Center, Nashville, Tennessee, United States of America; 7 Vanderbilt Ingram Cancer Center, Nashville, Tennessee, United States of America; 8 Veterans Affairs Tennessee Valley Health Care System, Nashville, Tennessee, United States of America; 9 Huntsman Cancer Institute, University of Utah, Salt Lake City, United States of America; National University of Singapore, Singapore

## Abstract

Myeloid translocation genes (MTGs) are transcriptional corepressors originally identified in acute myelogenous leukemia that have recently been linked to epithelial malignancy with non-synonymous mutations identified in both MTG8 and MTG16 in colon, breast, and lung carcinoma in addition to functioning as negative regulators of WNT and Notch signaling. A yeast two-hybrid approach was used to discover novel MTG binding partners. This screen identified the Zinc fingers, C2H2 and BTB domain containing (ZBTB) family members ZBTB4 and ZBTB38 as MTG16 interacting proteins. ZBTB4 is downregulated in breast cancer and modulates p53 responses. Because ZBTB33 (Kaiso), like MTG16, modulates Wnt signaling at the level of TCF4, and its deletion suppresses intestinal tumorigenesis in the *Apc^Min^* mouse, we determined that Kaiso also interacted with MTG16 to modulate transcription. The zinc finger domains of Kaiso as well as ZBTB4 and ZBTB38 bound MTG16 and the association with Kaiso was confirmed using co-immunoprecipitation. MTG family members were required to efficiently repress both a heterologous reporter construct containing Kaiso binding sites (4×KBS) and the known Kaiso target, Matrix metalloproteinase-7 (MMP-7/Matrilysin). Moreover, chromatin immunoprecipitation studies placed MTG16 in a complex occupying the Kaiso binding site on the *MMP-7* promoter. The presence of MTG16 in this complex, and its contributions to transcriptional repression both required Kaiso binding to its binding site on DNA, establishing MTG16-Kaiso binding as functionally relevant in Kaiso-dependent transcriptional repression. Examination of a large multi-stage CRC expression array dataset revealed patterns of *Kaiso*, *MTG16*, and *MMP-7* expression supporting the hypothesis that loss of either Kaiso or MTG16 can de-regulate a target promoter such as that of *MMP-7*. These findings provide new insights into the mechanisms of transcriptional control by ZBTB family members and broaden the scope of co-repressor functions for the MTG family, suggesting coordinate regulation of transcription by Kaiso/MTG complexes in cancer.

## Introduction

The myeloid translocation gene proteins (MTGs) are transcriptional corepressors, lacking both enzymatic and DNA-binding activities, that act as scaffolding proteins upon which other transcriptional corepressors (mSin3a, N-CoR, SMRT), histone deacetylases (HDACs), and transcription factors assemble [1], [2]. Their modular nature permits contributions to multiple promoter-specific repressor complexes, making them master regulators of gene expression. MTG8 and MTG16 (CBFA2T3) were originally identified as targets of chromosomal translocations in acute myeloid leukemia [1], [2], suggesting an important role in cellular growth and development. MTG family proteins have since been implicated in the development of colon and breast cancers as well [3], [4], [5], [6]. Because MTG repressive capabilities are dictated via protein-protein interactions with transcription factors and other co-repressors it is imperative to identify MTG-interacting proteins and understand their functions and specificities in the context of tumorigenesis.

Zinc fingers, C2H2 and BTB domain containing (ZBTB) family members bind methylated or unmethylated DNA in a sequence-specific manner and repress target genes [7]. A ZBTB family member that has recently been studied in detail for its ability to modulate colorectal cancer, Kaiso (ZBTB33), utilizes all three zinc fingers for high affinity binding to DNA targets [8]. All ZBTB family members likely share this method of DNA binding, as homology exists within their zinc finger motifs. Kaiso, like MTGs, forms repression complexes that include N-CoR and HDACs [1], [9] and plays a pivotal role in the repression of tumor suppression genes such as *CDKN2A* in colon cancer cell lines [10]. Kaiso also represses matrix metalloproteinase gene *MMP-*7 expression [11] in addition to canonical and non-canonical Wnt targets [12], [13], [14]. Moreover, Kaiso expression is a prognostic indicator in non-small cell lung cancer [14] and its deletion suppresses intestinal tumorigenesis in mice [15]. ZBTB4 also modulates the cellular responses to p53 activation [16] and is downregulated in breast cancer [17]. Collectively, these data suggest ZBTB proteins, and Kaiso specifically, have multidimensional roles in cancer development.

Like the ZBTB family of transcription factors, the MTG family of transcriptional corepressors has disparate roles in cancer development. For example, MTGs associate with TCF4 to repress Wnt signaling [18] and mutations in both MTG8 and MTG16 were found in colon and breast cancer [4], [5]. *Mtgr1* is required for tumorigenesis in a murine model of inflammatory carcinogenesis [6], and MTG16 has been identified as a putative tumor suppressor in human breast cancer [3]. Given that ZBTB16 (also known as PLZF) and BCL6 (ZBTB27) associate with MTG8 [19], [20] and that Kaiso and MTG16 have similar influences in cancer development, we tested for a wider structure-function relationship between the ZBTB and MTG families, which could provide insights into their roles in tumorigenesis.

Here, we identified the association of Kaiso, ZBTB4, and ZBTB38 with MTG16 in yeast two-hybrid assays, which suggests a direct physical interaction between these factors. The Kaiso-MTG16 complex specifically binds to Kaiso's established binding site (KBS) on DNA and enhances repression of a KBS-containing reporter and the Kaiso target, *MMP-7* promoter. However, MTG family members did not influence methylation-based repression by Kaiso. These data suggest that Kaiso-MTG16 dependent transcriptional repression requires the interaction of this complex on the KBS. Moreover, the impact of MTG16 on repression of Kaiso target promoters depends on Kaiso DNA binding. Analysis of publicly available ChIP-seq datasets showed that MTG family members bind Kaiso-targeted promoters over 70% of the time, implicating this interaction in the regulation of over 100 genes. Lastly we examined a large multi-stage CRC expression array dataset and discovered an inverse relationship between *Kaiso* and *MTG16* expression and consistently elevated *MMP-7* expression at all stages of tumorigenesis supporting the hypothesis that loss of either Kaiso or MTG16 de-regulates *MMP-7* expression.

## Materials and Methods

### Yeast Two-Hybrid

**Figure 1:**
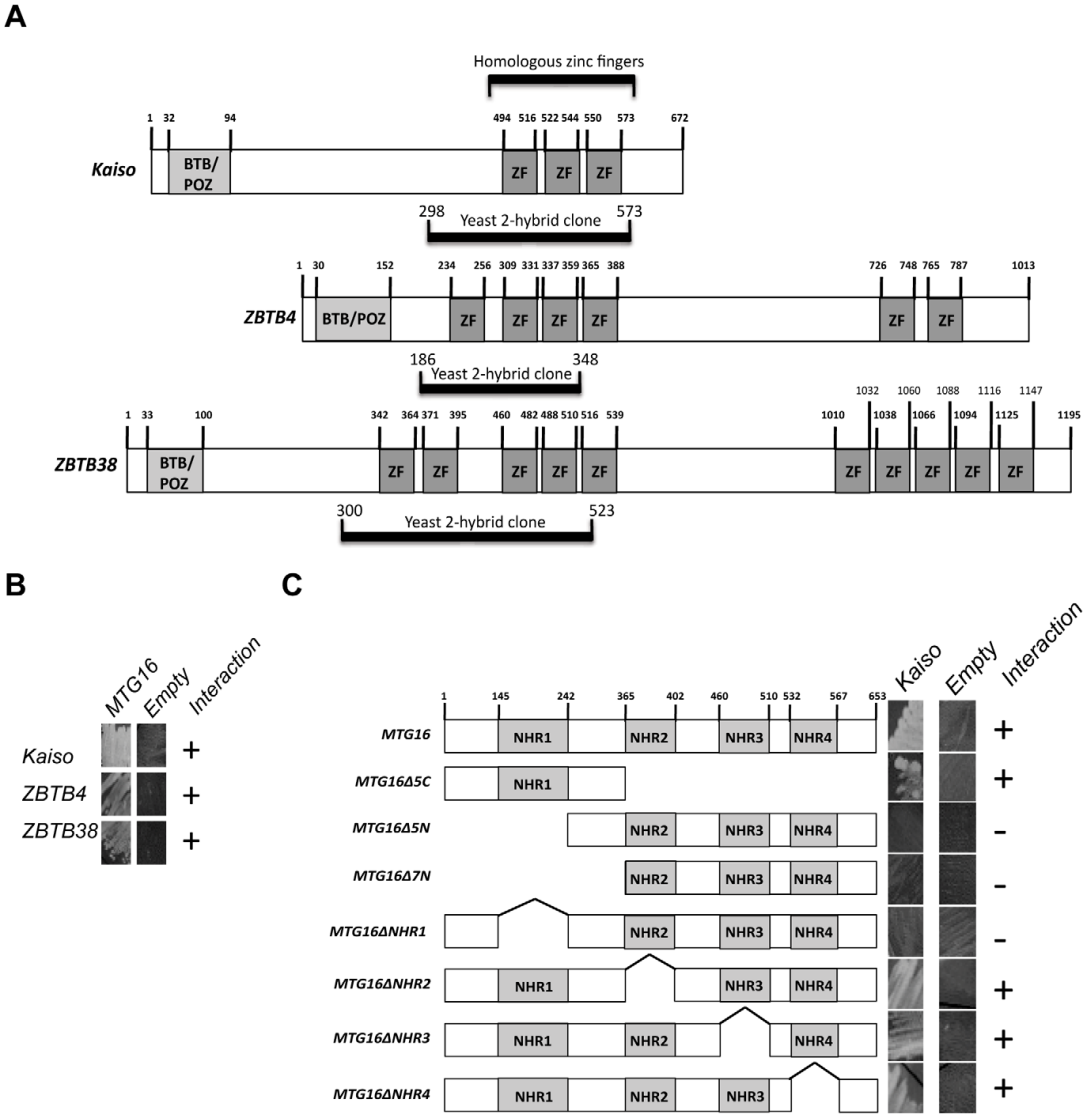
ZBTB family members interact with MTG16. A. ZBTB family member alignment based on homologous zinc fingers and identification of amino acids used in yeast-two hybrid experiments (yeast two-hybrid clones). B. Yeast-two hybrid assay for interactions between ZBTB family members and MTG16. C. Mapping of the Kaiso binding site on MTG16. Yeast two-hybrid assays were performed in triplicate.

*ZBTB4* and *ZBTB38* prey and *MTG16* bait plasmids were obtained from Hybrigenics. pDONR201-*Kaiso* (clone ID: HsCD00082434, The ORFeome Collaboration) and pDONR221-*MTG16* (clone ID: HsCD00079915, HIP) plasmids were obtained from Harvard PlasmID and pGAD-GW and pGBT9-GW vectors were provided by ABR. Kaiso and MTG16 were inserted into the pGAD-GW and pGBT9-GW plasmids using the Gateway Cloning procedure [21]. In short, 150 ng of entry vector (*Kaiso* or *MTG16*) was mixed with 150 ng of destination vector (pGAD or pGBT9) and the volume was brought to 8 µl with TE. 2 µl of LR Clonase II enzyme mix (Invitrogen) was added and the reaction was incubated for 1 hour at 25°C. 1 µl Proteinase K was then added to each sample and incubated at 37°C for 10 minutes. Kaiso was then truncated to include amino acids 298–573 by Agilent Quickchange II site-directed mutagenesis (Agilent) according to the manufacturer's protocol. The MTG16 constructs shown in Figure 1 were also developed using the Quickchange II site-directed mutagenesis kit. For the yeast two-hybrid, yeast at OD_600_ were transformed via the PEG-LiAc-TE method using a 42°C heat shock. Yeast at an OD_600_ of 0.6 were centrifuged at 3000 rpm for 5 minutes and resuspended in 10 ml of LiAc-TE, centrifuged and resuspended in 1 ml of LiAc-TE. Transformations were set up using 50 ug of carrier DNA (sheared salmon sperm DNA, Ambion), 1 ug of DNA for each construct (pGAD and pGBT9) and 100 ul of competent yeast. 0.7 ml of sterile 40%PEG-LiAc-TE was added and yeast were allowed to recover at 30°C for 30 minutes and then heat shocked at 42°C for 15 minutes. Cells were then washed with sterile TE and the reactions were plated on double-dropout selective media plates (-leu, -trp) and grown at 30°C for three days. Colonies were then plated on either triple- (for the Hybrigenics system) or quadruple-dropout (for the pGAD/pGBT9 system, -leu, -trp, -his, -ala) plates. Empty vector bait was used as a negative control.

### Immunofluorescence and Colocalization

K562 human leukemia cells (ATCC CCL-243) were plated on poly-l-lysine (Sigma-Aldrich) coated cover slips and allowed to adhere for 30 minutes at room temperature. Cells were fixed with ice-cold 2% paraformaldehyde, pH 7.4 for 10 minutes. After three washes, cells were blocked with 1% BSA and then stained with MTG16 antibody (1∶1000 333, supplied by MEE) and a polyclonal Kaiso antibody (1∶1000 supplied by ABR) overnight at 4°C. Cells were washed then stained with anti-rabbit-Cy3 and anti-mouse-FITC for 1 hour at room temperature and mounted using ProLong Gold antifade mounting medium with DAPI (Invitrogen). Slides were visualized using Deltavision software (Stress Photonics, Madison, WI) supplied by the Epithelial Biology Core (Vanderbilt).

### Cell Culture

Human colon cancer cell lines HCT116 (ATCC CCL-247) and HT29 (ATCC HTB-38) were maintained in McCoy's 5A media supplemented with fetal bovine serum (FBS) and pen/strep (P/S). HCT116 cells were transfected with Lipofectamine LTX and Plus reagent (Invitrogen) and HT29 cells were transfected with Fugene HD (Promega) according to manufacturer's protocol. K562 cells were maintained in DMEM supplemented with FBS and P/S.

### Coimmunoprecipitation

HCT116 cells were transfected with Kaiso and each of the Gal- or Myc-tagged MTG family members (provided by Mike Engel) using Lipofectamine LTX and Plus reagent (Invitrogen) according to manufacturer's protocol. 48 hours post-transfection, cells were lysed with coimmunoprecipitation buffer (0.5% Triton-X 100, 0.1% DOC, 0.1% SDS, and protease inhibitors in PBS), clarified by centrifugation, and pre-cleared with protein A/G agarose beads for 1 hour at 4°C. 2 ug of either anti-Gal (Santa-Cruz, sc-510), anti-Myc (Santa-Cruz, sc-40) or anti-IgG (Cell Signaling, G3A1) control antibodies were then added to pre-cleared lysate and incubated overnight at 4°C with rotation. Following removal of input, 40 µl protein A/G agarose beads were added to each sample and incubated at 4°C for 1 hour with rotation. Western blotting for Kaiso (polyclonal Kaiso antibody supplied by ABR, 1∶1000) was then performed.

### Kaiso Knockdown

HEK293T packaging cells (ATCC CRL-11268) were transfected with Mission shRNA constructs (Sigma-Aldrich) specific for human Kaiso (clone ID: NM_006777.3-2600s1c1 and NM_006777.3-358s1c1) as well as the PAX2 and MDG2 plasmids using Superfect Transfection Reagent (Qiagen) according to the manufacturer's protocol. 24 hours post-transfection, media was removed from the HEK293T cells and passed through a 0.2 µM filter. 4 µg/ml Polybrene (Millipore) was added to the filtered media. Growth medium was removed from HCT116 cells and replaced by infection medium. After 6 hours, the infection medium was replaced with normal growth medium and the infection process was repeated 24 hours later. Cells were selected for knockdown using 5 µg/ml Puromycin (Invitrogen) over a 3-day period. Knockdown was analyzed by the delta-delta-CT method following RT-PCR using Kaiso-specific Taqman probes (Invitrogen) and by Western blotting using the polyclonal Kaiso antibody (1∶1000, supplied by ABR).

### Luciferase Reporter Assays

Kaiso and MTG family members were transiently expressed in HCT116 cells along with either the Kaiso binding site (4×KBS) reporter, which contains four consensus Kaiso binding sites and a luciferase reporter gene (gift from Juliet Daniel, McMaster University, Canada), or the mutant Kaiso binding site promoter which contains mutant forms of the KBS and a luciferase reporter gene (PGL3 control 4×KBSMT, gift from Juliet Daniel). A constitutive reporter plasmid expressing Renilla luciferase (pGL4 TK hRluc) was included to normalize data generated from the Kaiso reporter constructs. Cell lysates were subjected to a dual-luciferase assay and presented as adjusted RLUs. For human *MMP-7* (HMAT, gift from BMF) assays, Myc-tagged delta-MTG16 constructs (ME, Figure 1C) were co-transfected with activators PEA3, LEF1, C-JUN, and delta β-Catenin and the human *MMP-7* promoter fused to the luciferase reporter gene (HMAT-2.3). Activation by enforced expression of PEA3, LEF1, C-JUN, and delta β-catenin was required to see repression by MTG family members because HCT116 cells express low levels of *MMP-7* endogenously. *Mbd2^−/−^* cells (a gift from Pierre-Antoine Defossez) [22] were maintained in Advanced MEM supplemented with BCS and P/S and transfected with Superfect (Qiagen), according to manufacturer's protocol. For methylation-dependent studies, 5 µg of pSV40Luc was methylated upon mixture of 1.5 µl SAM, 30 µl 10× NEB2, 1 µl SssI methylase (New England Biolabs) and water to 300 µl. The mixture was incubated at 37°C for 2 hours followed by addition of 1.5 µl SAM and continued incubation for 1 hour. DNA was then purified using the Qiaquick PCR purification kit (Qiagen). *Mbd2^−/−^* cells (gift from Pierre-Antoine Defossez) were transfected with MTG family members and either methylated or unmethylated promoter according to previously published specifications [22]. HMAT and KBS assays were performed as outlined in the Materials and Methods section in the main paper.

### MMP-7 Repression

HT29 cells were transfected with expression plasmids for Kaiso, an MTG family member, or a combination of the two. RNA was isolated using the Qiagen RNA Isolation Kit and cDNA was generated from 1 µg of total RNA using the iScript cDNA Synthesis Kit (BioRad). Taqman qPCR was performed using *MMP-7* specific probes (Invitrogen), and expression was normalized to *GAPDH* (Invitrogen). Analysis was performed using the delta-delta Ct method. Transfected cells were also treated with Protein transport inhibitor containing Brefeldin A (BD GolgiPlug) for 3 hours before lysate was collected using RIPA buffer. Lysates were run on Western blots, probed with α-MMP-7 (Santa-Cruz 1∶1000) and α-β-actin (Sigma-Aldrich, 1∶5000) as a loading control and developed using the Odyssey system.

### Chromatin Immunoprecitation

HCT116 cells were fixed using 1% formaldehyde for 20 minutes at room temperature. Cells were then lysed and chromatin was sheared by sonication (4 watts, 10 seconds on, 1 minute off, on ice repeated ten times). Lysate was precleared with 20 ul protein A/G agarose beads (Santa Cruz, sc-2003) and beads were blocked with 2 ug sheared salmon sperm DNA (Ambion). Lysate was immunoprecipitated with either IgG (Cell Signaling, G3A1), polyclonal MTG16 (333, provided by MEE) or monoclonal MTG16 (2D1, provided by SWH) overnight at 4°C with rotation. Protein A/G agarose beads were then added for 1 hour at 4°C with rotation. Complexes were eluted and crosslinks reversed with NaCl at 65 degrees for 5 hours and DNA isolated using the Qiagen PCR purification kit. qRT-PCR was performed using Kaiso binding site-specific primers [11]. Analysis was performed using the delta-delta Ct method.

### Statistical Methods

Luciferase, ChIP, and expression analyses were analyzed using one-way ANOVA and a Newman-Keuls post-test in Graphpad Prism 5.0c, unless otherwise indicated.

### Microarray experiments—human tissues and microarray platform

Representative sections of fresh tissue specimens were flash frozen in liquid nitrogen and stored at −80C until RNA isolation. Quality assessment slides were obtained to verify the diagnosis of cancer or normal adjacent mucosa. Stage was assessed using American Joint Commission on Cancer guidelines for both cohorts of tumor samples. RNA for human tissue was purified using the RNeasy kit (Qiagen). Samples were hybridized to the Human Genome U133 Plus 2.0 GeneChip Expression Affymetrix array. The expression array data are deposited and the GEO accession number is GSE17538. Complete minimum information about a microarray experiment-compliant dataset for analysis are available (http://www.ncbi.nlm.nih.gov/geo/query/acc.cgi?acc=GSE17538).

### Microarray Analysis and Statistics

Microarray data were normalized using the Robust MultiChip Averaging (RMA) algorithm as implemented in the Bioconductor package *Affy* as previously described [23], [24], [25]. Wilcoxon Rank sum test was used to determine significance for the normal, adenoma and colorectal cancer comparisons. The gene expression data Spearman correlation coefficient was used to assess the association between *Kaiso* and *MTG16* (probes 214631_at and 208056_s_at respectively). We next compared the differential expression between normal samples and carcinoma samples for genes MTG16 and *MMP-7* using a linear model with an interaction term. More specifically, our model included gene expression as the outcome variable, main effects group (normal, carcinoma) and gene (e.g., *Kaiso, MMP-7*) as well as the group×gene interaction term. The significance of interaction term indicates that differential gene expression for normal vs. carcinoma is significantly different for the two genes. This test procedure was similarly applied to genes *Kaiso* versus *MMP-7*.

### Ethics Statement

The protocols and procedures for this study were approved by the Institutional Review Boards at University of Alabama-Birmingham Medical Center, Vanderbilt University Medical Center, Veterans Administration Hospital (Nashville, TN) and H. Lee Moffitt Cancer Center, and written informed consent was obtained from each subject per institutional protocol.

## Results

### ZBTB family members interact with MTG16

In order to understand MTG16-mediated intracellular signaling we used yeast-two hybrid screening to identify MTG16 binding partners. This approach identified two members of a family of zinc-finger transcription factors that repress methylated and non-methylated DNA: ZBTB4 and ZBTB38. Another member of this family, Kaiso (ZBTB33), is of particular interest because, like MTG16, it has been previously linked to Wnt signaling and tumorigenesis in the gut [10], [12], [15]. Kaiso was not identified in the initial yeast two-hybrid screen, likely due to limited expression in the murine brain library that was screened. However, the ZBTB4 and ZBTB38 clones that were identified consisted of the highly homologous zinc finger region shared by ZBTB family members (Fig. 1A, S1) and suggested that Kaiso would likely be a third target for MTG16 binding. Directed yeast two-hybrid validation experiments demonstrated that ZBTB4, ZBTB38, and Kaiso each interact with MTG16 via a highly conserved zinc-finger domain (Fig. 1B) which is also required for ZBTB16 (PLZF) MTG16 interaction [19], but not with ZBTB27 (BCL6) which demonstrates MTG16 binding within the fourth zinc finger motif [20](Fig. S1). Mapping of the MTG16 binding domain identified the nervy homology region 1 (NHR1) as the component of MTG16 that is required for binding to Kaiso as the three constructs lacking the NHR1 domain (Δ5N, Δ7N, and ΔNHR1) failed to demonstrate an interaction in the yeast two-hybrid assay (Fig. 1C).

**Figure 2:**
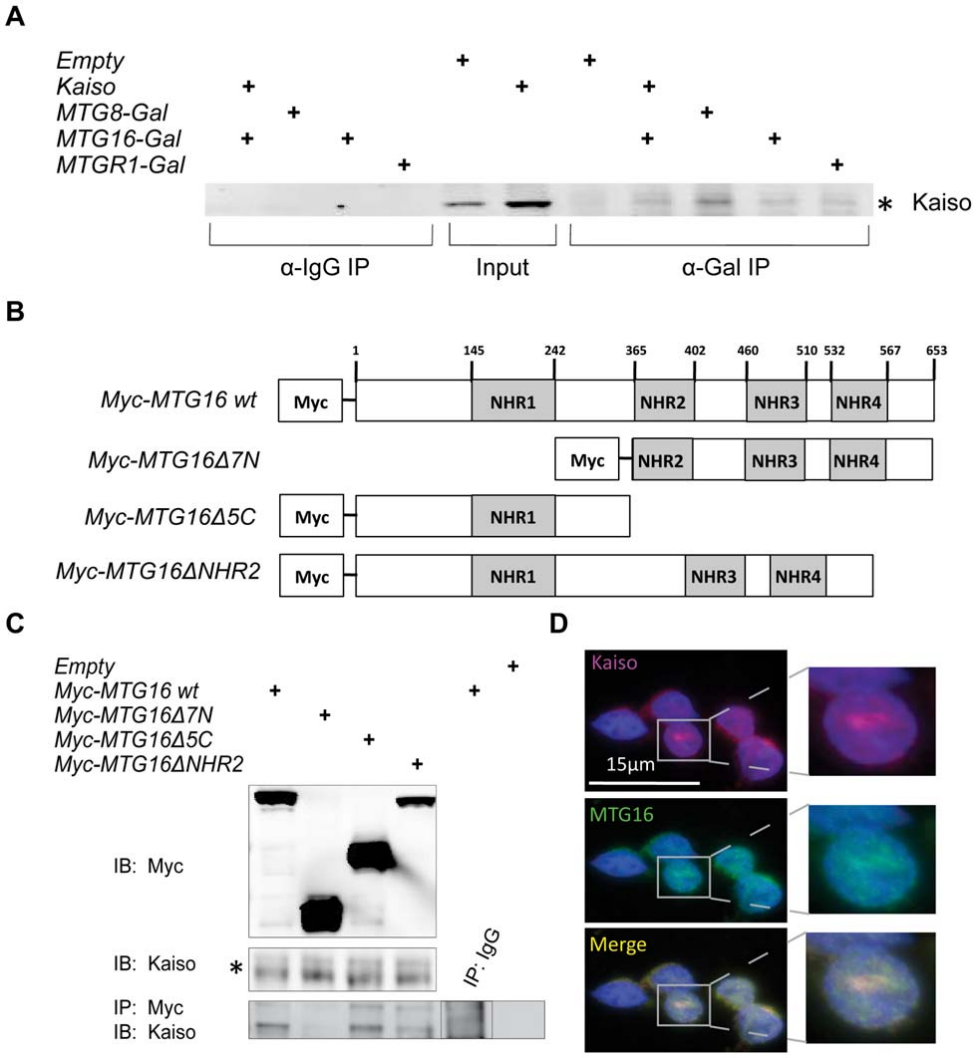
Kaiso and MTG16 associate and co-localize. A. Immunoprecipitation of overexpressed Gal-tagged MTG family members in HCT116 cells. Cell lysates were fractionated and immunoprecipitated with anti-Gal or control IgG and analyzed by immunoblotting with Kaiso antibody. B. Myc-tagged MTG16 constructs used for immunoprecipitation mapping of MTG16 binding site. C. Immunoprecipitation of overexpressed Myc-tagged MTG family members in HCT116 cells. Cell lysates were fractionated and immunoprecipitated with anti-Myc or control IgG and analyzed by immunoblotting with Kaiso antibody. D. K562 cells costained for MTG16 (333, FITC) and Kaiso (polyclonal, ABR, Cy3) were imaged using the Deltavision system, and a correlation coefficient was determined for Kaiso and MTG16 colocalization (r = 0.95±0.04, n = 25 cells).

Using wild-type and selected mutant constructs for MTG16, we confirmed the Kaiso interaction in the HCT116 human colon cancer cell line which has endogenous Kaiso expression (Fig. 2A, r = 0.95±0.04). We then mapped the binding interface to the N-terminus of MTG16 (Fig. 2B,C). We next used immunofluorescence to demonstrate endogenous co-localization of Kaiso and MTG16 in the K562 cells, a human leukemia cell line with high levels of expression of both proteins (Fig. 2D). Furthermore, *in silico* analysis of publicly available ChIP-seq data (UCSC Genome Browser, [26]) identified 101 promoters with intersecting Kaiso and MTG protein occupancy (Fig. S2A), providing further evidence that cooperativity between Kaiso and the MTG proteins may occur. Furthermore, three targets identified in the *in silico* ChIP-seq analysis, *ATF-2*, *ATF-7*, and *MAPK14* demonstrate altered expression in response to MTG16 overexpression. *ATF-2* and *MAPK14* are repressed by MTG16 (Fig. S2B, *P*<0.0001 *ATF-2* and *P* = 0.04 *MAPK14*) while *ATF-7* expression is higher in response to MTG16 overexpression (Fig. S2C, *P*<0.0001). Though initially surprising, this result still points to a Kaiso and MTG16 complex binding the *ATF-7* promoter but suggests that either the proteins behave as activators, something Kaiso is capable of on this promoter, or that they are displacing another repressor complex that is a more potent repressor than the Kaiso-MTG16 complex. Collectively, these findings suggest a direct binding relationship between Kaiso and MTG16 with a shared impact in transcriptional control and tumorigenesis.

### MTG family members repress the Kaiso binding site promoter

**Figure 3:**
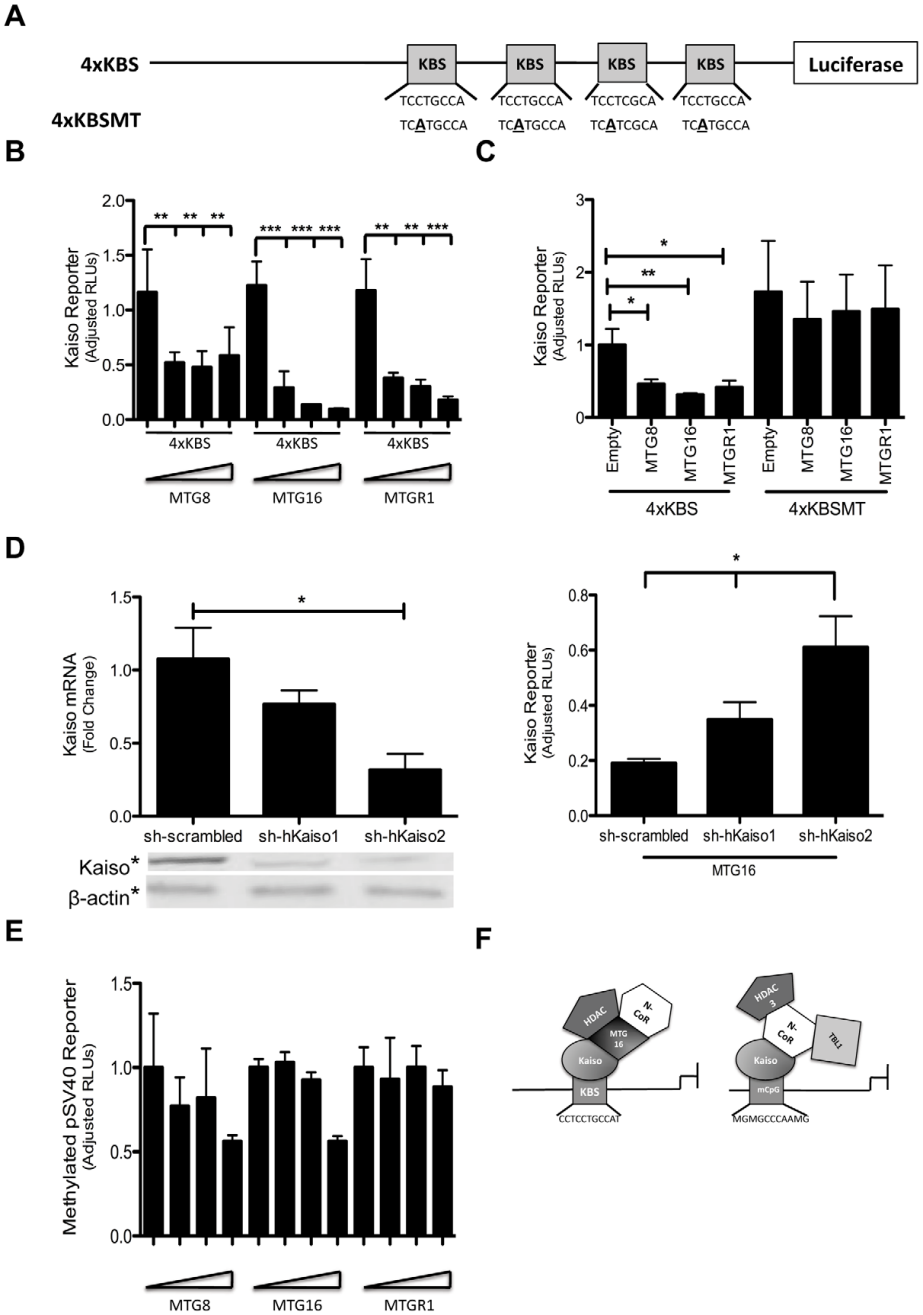
MTG family members repress the Kaiso binding site reporter (4×KBS). A. Composition of the KBS reporter constructs. The 4×KBS reporter contains four consensus Kaiso binding site sequences followed by a luciferase gene. The mutant reporter (4×KBSMT) contains a C→A transversion in the Kaiso binding sites which abolishes Kaiso binding. B. 4×KBS artificial promoter assays in HCT116 cells upon titration of MTG family members (0 ng, 200 ng, 400 ng, 800 ng). The graph shows the fold-change in luciferase activity relative to the control reporter, pGL4-TK hRLUC, after transfection of expression plasmids encoding MTG family members, MTG8, MTG16, and MTGR1. The error bars represent the standard error of the mean for 3 replicate experiments performed in triplicate. C. 4×KBS and 4×KBSMT artificial promoter assays in HCT116 cells after transfection of 500 ng of the indicated MTG family member. The error bars represent the standard error of the mean for 4 replicate experiments performed in triplicate. D. Kaiso knockdown by two independent Kaiso shRNA constructs compared to a scrambled shRNA control (left). 4×KBS artificial promoter assay after knockdown of Kaiso and the transfection of 500 ng of MTG16 (right). E. Methylated pSV40 artificial promoter assay in *Mbd2^−/−^* cells upon titration of MTG family members (0 ng, 200 ng, 400 ng, 800 ng). The graph shows the fold-change in luciferase activity relative to the control reporter, pGL4-TK hRLUC, after transfection of expression plasmids encoding MTG family members, MTG8, MTG16, and MTGR1. The error bars represent the standard error of the mean for 3 replicate experiments performed in triplicate. F. Model for Kaiso repression of Kaiso binding site (KBS) and methyl CpGs (mCpG) on target promoters. *P<0.05, **P<0.01, ***P<0.001.

In mammalian cells, Kaiso recognizes both the consensus sequence TCCTGCNA (Kaiso binding site, KBS) and methylated CpG dinucleotides [22], [27]. We used a luciferase-based Kaiso binding site reporter (4×KBS), as well as a mutant form that contains a C→A transversion that abolishes both Kaiso binding and Kaiso-mediated repression (4×KBSMT) [27] to explore cooperation between Kaiso and MTG family proteins in transcriptional repression (Fig. 3A). We determined that MTGs were capable of repressing the 4×KBS reporter construct (Fig. 3B) and that this repression was specific as it was disrupted when the 4×KBSMT reporter was used in lieu of the 4×KBS reporter (Fig. 3C). Additionally, Kaiso knockdown (Fig. 3D, left) resulted in a dose-dependent loss of 4×KBS repression by MTG16 (Fig. 3D, right). Finally, knockdown of both MTG16 and Kaiso resulted in decreased repression of the KBS in the HCT 116 cell line that expresses high levels of both proteins (Fig. S3A & B, *P* = 0.008).

Because we determined MTG family members are able to repress the sequence-specific Kaiso target, we also wanted to determine whether MTGs were able to repress the second common Kaiso target, methyl-CpG. We therefore assessed the ability of MTG family members to repress a methylated reporter plasmid [22]. In this case, we saw little repression of the methylated reporter by MTG family members until very high levels of MTG8 or MTG16 were used (Fig. 3E). This indicates that Kaiso dependent MTG repression specificity is directed towards genes containing the KBS as opposed to methyl-CpG, and suggests a model where MTG16 cooperates with Kaiso in the repression of KBS targets, but not to methylated CpG targets (Fig. 3F).

### MTG family members repress the *MMP-7* promoter

**Figure 4:**
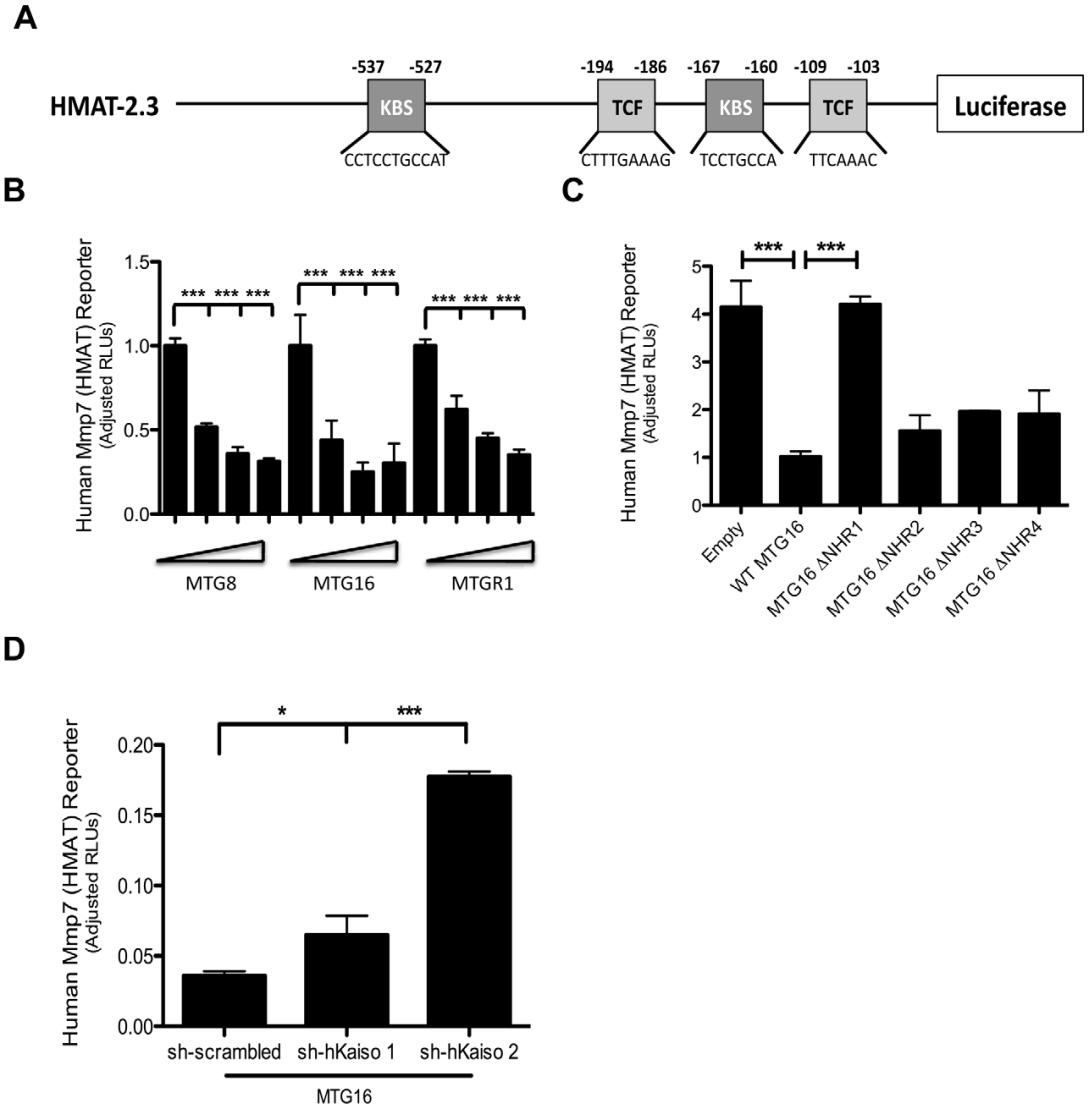
MTG family members depend on Kaiso to repress the human *MMP-7* promoter. A. The composition of the full-length *MMP-7* reporter (HMAT-2.3). The HMAT-2.3 reporter contains two Kaiso and two TCF binding sites followed by a luciferase gene. B. HMAT-2.3 artificial promoter assays in HCT116 cells upon titration of MTG family members (0 ng, 200 ng, 400 ng, 800 ng). The graph shows the fold-change in luciferase activity relative to the control reporter, pGL4-TK hRLUC, after transfection of expression plasmids encoding MTG family members, MTG8, MTG16, and MTGR1. The error bars represent the standard error of the mean for 3 replicate experiments performed in triplicate. C. HMAT-2.3 promoter assay utilizing MTG16 deletion constructs in which the indicated nervy homology region is removed. Error bars represent the standard deviation of triplicate samples. D. HMAT-2.3 artificial promoter assay after knockdown of Kaiso and the transfection of 500 ng of MTG16. Error bars represent the standard deviation of triplicate samples. *P<0.05, ***P<0.001.

MMP-7 is an established tumor promoter in colon, prostate, pancreatic, and lung cancers [28], [29], [30], [31]. It also contributes to tumor progression and metastasis [32]. Interestingly, two Kaiso binding sites are present in the human *MMP-7* promoter (Fig. 4A) that enable Kaiso-mediated repression of MMP-7 expression [27]. Our data suggests that MTGs may cooperate with Kaiso to repress Kaiso targets. Therefore, we hypothesized that MTGs would repress the *MMP-7* promoter. Titration of each MTG family member with the full-length human *MMP-7* reporter (HMAT-2.3, [27]) in the Kaiso-expressing HCT116 cells resulted in dose-dependent repression (Fig. 4B). This repression was lost when an MTG16 construct lacking the NHR1 domain (loss of affinity mutant) was used, further supporting an interaction with Kaiso that is dependent on the NHR1 domain of MTG16 (Fig. 4C). Also, Kaiso knockdown resulted in loss of HMAT repression, suggesting that Kaiso represses *MMP-7* via MTG16 recruitment (Fig. 3D & 4D). Moreover, knockdown of both MTG16 and Kaiso in the HCT116 cell line resulted in decreased repression of the HMAT reporter (Fig. S3A & C, *P*<0.0001). Taken together, these data demonstrate that MTG16 and Kaiso repress *MMP-7* promoter activity.

**Figure 5:**
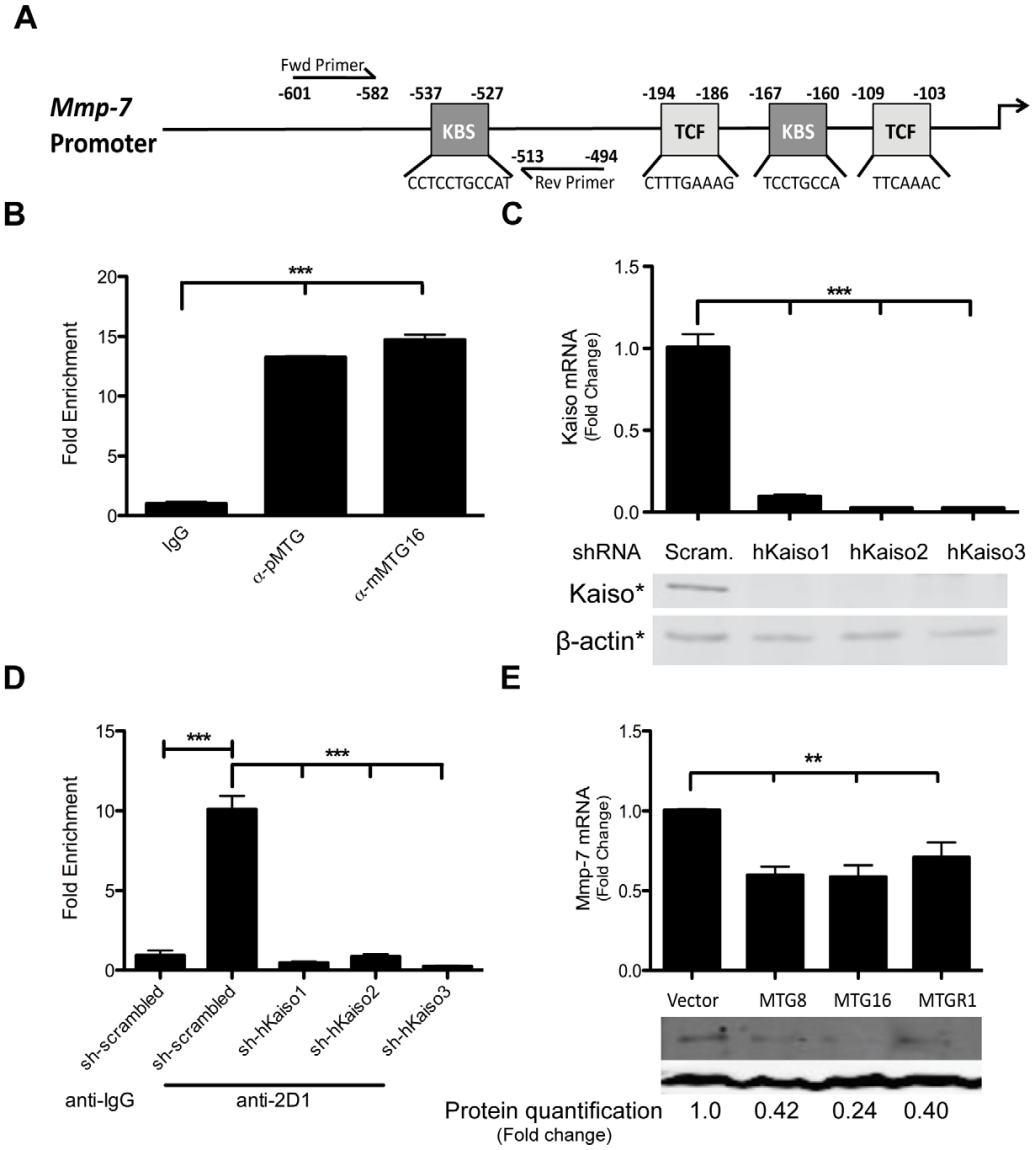
MTG family members repress endogenous *MMP-7*. A. Primers for the ChIP assay flank the −537 Kaiso binding site in the *MMP-7* promoter. B. ChIP assay performed in HCT116 cells using both polyclonal (333, pMTG) and monoclonal (2D1, mMTG16) antibodies for MTG16. The graph shows fold-enrichment compared to IgG control. C. Knockdown of Kaiso by three independent shRNA constructs compared to a scrambled sh-RNA control. D. Chromatin immunoprecipitation assay in HCT116 cells. The graph shows fold enrichment of the −537 Kaiso binding site after precipitation with the MTG16 2D1 antibody compared to the IgG control. E. *MMP-7* mRNA expression after transfection of the indicated MTG family member in HT29 cells. The graph shows the fold-change (ΔΔCt) of *MMP-7* mRNA compared to an empty control. Error bars represent the standard error for three replicate experiments performed in triplicate (top). Representative immunoblotting of MMP-7 in HT29 cells after transfection of indicated family members and treatment with Brefeldin-A. Protein quantification was performed with the Odyssey Western blot developer system. **P<0.01, ***P<0.001.

The human *MMP-7* promoter also contains two TCF binding sites (Fig. 5A). MTGs also associate with TCF4 and repress TCF4 targets [18]. Thus it was possible that in addition to repressing via an interaction with Kaiso at the KBS, MTG16 could be recruited by TCF4 to repress *MMP-7* via TCF response elements. To address this issue of MTG16 specificity for Kaiso or TCF binding sites, we performed repression assays using heterologous transcriptional reporters composed of minimal *MMP-7* promoter elements where TCF binding sites were mutated individually or in combination [33] (Fig. S4A). Mutation of either or both of these sites had no effect on MTG16-mediated repression of *MMP-7* (Fig. S4B). These data indicate that MTG16-dependent *MMP-7* repression occurs independent of TCF binding sites. In concert, these results suggest that MTG16 recruitment by Kaiso to the *MMP-7* KBS is responsible for MTG16:Kaiso mediated *MMP-7* repression.

Both MTG16 and Kaiso have been implicated in breast, colon, and lung carcinoma [3], [4], [5], [10], [34], [35]. Each can regulate Wnt signaling [12], [18], a pathway strongly associated with colon cancer development. Recently at least five MTG mutations have been identified in colon or breast cancer [4], [5], all are non-synonymous and predicted to influence MTG function. We therefore wanted to determine whether these mutations could influence Kaiso dependent repression of the 4×KBS and HMAT reporters. Overexpression of each MTG mutant resulted in no change in repression of either the 4×KBS (Fig. S5A) or HMAT reporters (Fig. S5B), indicating that these mutations do not influence MTG dependent Kaiso repression.

### MTG family members repress endogenous MMP-7 expression

Based upon the transcriptional control data described above, we predicted that MTG16 should occupy the promoters of Kaiso-regulated genes. To address this hypothesis, we used ChIP to probe for MTG16 occupancy of the −537 Kaiso binding site in the endogenous *MMP-7* promoter (Fig. 5A). We observed 13.2 or 14.7 fold enrichment over IgG when either a polyclonal (333, pMTG) or monoclonal MTG16 (2D1, mMTG16) antibody was used (*P*<0.001, Fig. 5B). Importantly, Kaiso knockdown abrogated MTG16 binding to the *MMP-7* promoter, showing that Kaiso is essential for MTG16 recruitment (Fig. 5C, D).

We next determined whether MTG16 was capable of regulating endogenous *MMP-7* expression by analyzing *MMP-7* mRNA and protein levels in the context of enforced expression of MTG family members. For this experiment, the colon cancer cell line HT29 was utilized. We chose HT29 cells because they express and secrete high levels of MMP-7 and express little MTG16 compared to other colon cancer cell lines, making them ideal for establishing repression of endogenous MMP-7 (Fig. S6A, B). Overexpression of MTG8, MTG16, and MTGR1 resulted in decreased MMP-7 both at the RNA and protein levels when compared to a vector control (fold-change mRNA expression: 0.60 S.E. 0.05 (MTG8), 0.59 S.E. 0.07 (MTG16), 0.71 S.E. 0.09 (MTGR1), *P*<0.01 for each, Fig. 5E), and this data was confirmed in a non-cancer cell line, Cos7 (Fig. S7). Considering that HT29 transfection efficiency in these experiments was 44.85%±6.553, the data likely under-represent the impact of MTGs on MMP-7 expression. Moreover, knockdown of Kaiso and MTG16 in HCT116 cells resulted in derepression of endogenous MMP-7 expression (Fig. S3D, *P* = 0.04). These findings suggest that MTGs repress endogenous *MMP-7* expression via interaction with Kaiso.

### *MMP-7* increased expression is associated with decoupling of the MTG16 and Kaiso regulatory axis

**Figure 6:**
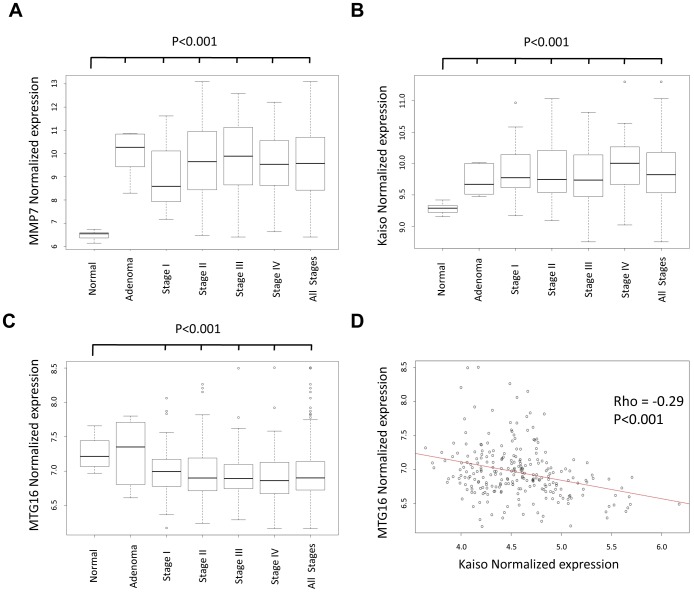
*MTG16* and *Kaiso* are negatively correlated in human CRC patient samples and *MMP-7* is up-regulated. A) Kaiso expression is significantly up-regulated in adenomas and colorectal cancer patients compared with normal adjacent colon tissues (*P*<0.001, for all comparisons). B) *MMP-7* expression is significantly up-regulated in adenoma and colorectal cancer patients for each stage (*P*<0.001 for all comparisons). C) *MTG16* expression is significantly down-regulated in colorectal cancers versus normal adjacent samples (*P*<0.001; *for all comparisons except adenoma). Wilcoxon Rank Sum test was used to determine significance for A–C. D) Normalized expression of both *MTG16* and *Kaiso* probes (see Methods) were compared in human colorectal cancer patients. The graph demonstrates a significant inverse correlation for *MTG16* versus *Kaiso* (*rho* = −0.29, *P*<0.001, inverse correlation depicted by red line, Spearman correlation coefficient).

We next sought to compare the expression levels of *Kaiso*, *MMP-7* and *MTG16* in patients with colorectal cancer compared with normal adjacent colon tissues and adenomas. We observed a significant up-regulation of *MMP-7* and *Kaiso* (Fig. 6A, B, *P*<0.001 for all stages) while *MTG16* is significantly down-regulated for all stages of cancer compared with normal adjacent colon samples (Fig. 6C, P<0.001). Given the *in vitro* data demonstrating MTG16 played a key role and was required for maximal repression of Kaiso-regulated KBS-containing promoters, we reasoned that Kaiso and MTG16 expression would be inversely related on a per sample basis. We did in fact note a highly significant inverse correlation (Fig. 6D, Rho = −0.29, *P*<0.001). The data presented in this paper suggests a model by which both Kaiso and MTG16 are necessary for *MMP-7* repression. This model likely applies to other Kaiso:MTG16 regulated KBS containing promoters.

## Discussion

Kaiso and MTG16 share several functional similarities, including their abilities to repress Wnt signaling, bind co-repressors, and impact processes that influence tumorigenesis. Because of these similarities, we postulated that Kaiso and MTGs could form complexes and that Kaiso could target MTG repression functions to specific promoters. In testing this hypothesis we found that Kaiso and MTG16 interact and that this depends on the homologous zinc fingers in Kaiso and the NHR1 domain in MTG16. Furthermore, when MTG family members are overexpressed, they enhance repression of a KBS reporter. Studies with both heterologous KBS reporters and Kaiso knockdown showed that MTG-dependent transcriptional repression on Kaiso targets, such as the *MMP-7* promoter, is Kaiso-dependent. ChIP experiments revealed that the Kaiso-MTG interaction occurs endogenously at the KBS in the *MMP-7* promoter, and reduced MMP-7 expression at both the RNA and protein level show the interaction to be functionally significant. Thus, we have linked two families of transcriptional repressors, both previously implicated in oncogenesis. Understanding the nature of this relationship is important in understanding transcriptional regulation by these complexes in normal biology and how this is perturbed in tumorigenesis.

Kaiso is a dual-specificity repressor that can recognize both a consensus sequence (KBS, TCCTGCNA) and methyl-CpG dinucleotides [27]. Interestingly, though MTG16 is important for repression of KBS reporters, it does not repress a methylated reporter, suggesting that the Kaiso-MTG16 interaction is specific for promoters that contain the Kaiso binding site. Kaiso has been identified as the factor that recruits the N-CoR complex to CpG-rich sequences in a methylation-dependent manner [36], our study demonstrates that there is a converse activity with interaction with MTG16, as it is a Kaiso cofactor that is dependent solely on Kaiso binding to the canonical KBS. This suggests that Kaiso might differentiate between target repression based on the availability of cofactors, making these cofactors key in understanding the regulation of Kaiso targets, be they methylated or the canonical consensus sequence. We think that this is biologically relevant as we noted a strong inverse correlation between *MTG16* and *Kaiso* levels in a large human CRC dataset. This could have important implications for the regulation of Kaiso:MTG16 targets and may influence the bimodal effect of Kaiso on carcinogenesis [10], [12], [15], [35].

This interaction is also of great importance as our data directly implicates MTG16:Kaiso complexes in the regulation of *MMP-7* expression. MMP-7 plays a significant role in all stages of tumor progression and is a key player in a wide variety of cancers including, but not limited to, colon, prostate, lung and ovarian [29], [37], [38], [39], [40]. Indeed, metalloproteinases are among the most common targets for anti-cancer drug development [41] so understanding their regulation will give insight into mechanisms by which their expression might be altered. We have determined that MTG16 binds Kaiso at the *MMP-7* promoter and recruits transcriptional corepressors to attenuate *MMP-7* expression. Furthermore, both *MTG16* and *Kaiso* demonstrate coordinate patterns of expression when compared with *MMP-7* in human CRC samples vs. normal adjacent colon samples. Decreased repression upon loss of either protein within this complex could contribute to tumorigenesis.

In order to better understand the scope of the Kaiso-MTG interaction, we analyzed public-domain ChIP-seq datasets (UCSC Genome Browser, [26]) to determine the overlap of targets bound by Kaiso and the MTG family members MTG16 and MTGR1 (Fig. S2A). Several targets relevant to cancer pathogenesis were revealed using this methodology. For example, Kaiso and MTG16 bind the Activating Transcription Factor *(ATF)-2* promoter, while Kaiso and MTGR1 bind the promoter for *ATF7*. The ATF family is composed of transcription factors that are involved in cellular stress response [42]. Aside from being activated by the MAPK pathway in response to stress, ATFs 2 and 7 are also involved in oncogenesis, as they are able to dimerize with c-Jun to influence Jun-dependent survival, apoptosis, and cell cycle progression [43], [44]. C-Jun pathway regulation is also seen with a third target of MTG-Kaiso binding, MAPK14. Mitogen-activated protein kinase (MAPK) p38α (MAPK14) suppresses cellular proliferation through inhibition of the JNK-c-Jun pathway [45] and plays an essential role in stem and progenitor cell proliferation and differentiation in the lung [46]. Another MAPK family member, MAP3K7, was also identified in this screen. MAP3K7 plays an important role in the modulation of TGF-β signaling [47], a pathway highly related to oncogenesis. Importantly, overexpression of MTG16 in the HT29 cell line resulted in repression of both *ATF2* and *MAPK14*, consistent with MTG16 functioning as a repressor, but also overexpression of the target *ATF7*, suggesting that this target has more complex transcriptional regulation. (Fig. S2B & C).

In summary, we identified an association between Kaiso and MTG family members. Because Kaiso is the prototypic ZBTB family member, we focused on understanding the contribution of MTG16 to Kaiso mediated repression. We determined that the Kaiso-MTG16 complex specifically binds to the KBS and represses the Kaiso target, *MMP-7*. We examined a large multi-stage CRC expression array dataset and discovered an inverse relationship between Kaiso and MTG16 expression and consistently elevated *MMP-7* expression at all stages of tumorigenesis supporting the hypothesis that loss of either Kaiso or MTG16 de-regulates *MMP-7* expression. Analysis of publicly available ChIP-seq datasets showed that MTG family members bind Kaiso-targeted promoters over 70% of the time, implicating this interaction in the regulation of over 100 genes, many of which are involved in cell cycle control and survival programs.

In conclusion, we report that Kaiso and MTGs interact in inhibitory complexes and identify a subset of target genes which are important in oncogenesis. Additional targets identified in the ChIP-seq screen include stress and oxidative damage response proteins potentially important in inflammatory carcinogenesis. Future experiments should be directed towards understanding the role of Kaiso and MTG16 in sporadic and inflammatory colon carcinogenesis.

## Supporting Information

Figure S1 **ZBTB family members interact with Kaiso through their homologous zinc finger domains.** Uniprot alignment between the five ZBTB family members known to bind MTG16 indicates homology within the zinc fingers (highlighted in purple) and BTB (highlighted in green) domains of each protein (white indicates 0% homology and dark blue indicates 100% homology). Yeast-two hybrid assay indicates binding of Kaiso (orange underline) ZBTB4 (black underline) and ZBTB38 (red underline) to MTG16 within the homologous zinc finger region in a similar manner to ZBTB16 (green underline).
(TIF)

Figure S2 **Kaiso shares repression targets with MTG16 and MTGR1.** A. The UCSC genome browser ChIP-seq data was used to identify Kaiso targets. Chip-seq data developed by Soler et al., 2011 [26] was used to identify MTG16 and MTGR1 targets that overlapped with Kaiso binding sites. B. *ATF-2*, *MAPK14*, and C. *ATF-7* mRNA expression upon overexpression of MTG16. The graph shows the fold-change (^ΔΔ^Ct) of mRNA compared to an empty vector control. Error bars represent the standard error for three replicate experiments performed in triplicate. *P<0.05, ***P<0.001.
(TIF)

Figure S3 **Repression of 4×KBS, HMAT, and** ***MMP-7*** **is decreased with knockdown of MTG16 and Kaiso in HCT116 cells.** A. *Kaiso* or *MTG16* mRNA expression after knockdown of Kaiso (sh-Kaiso) and MTG16 (si-MTG16) in HCT116 cells. The graph shows the fold-change (^ΔΔ^Ct) of mRNA compared to a scrambled control (sh-scrambled and si-scrambled). B. 4×KBS and C. HMAT reporter activity after knockdown of both Kaiso and MTG16. D. *MMP-7* expression in response to knockdown of Kaiso and MTG16. Error bars represent the standard error for three replicate experiments performed in triplicate. **P<0.01, ***P<0.001.
(TIF)

Figure S4 **MTG16 does not repress the** ***MMP-7*** **reporter via TCF binding sites.** A. The composition of the truncated *MMP-7* reporter (HMAT-296) and TCF mutant reporters (HMAT-194, HMAT-109, and HMAT-2×). B. HMAT artificial promoter assays in HCT116 cells. HCT116 cells were transfected with either 500 ng of MTG16 or 500 ng of Empty vector as a control. The graph shows the fold-change in luciferase activity relative to the standard pGL4-TK hRLUC after transfection of expression plasmids. The error bars represent the standard error of four replicate experiments performed in triplicate. *P<0.05, **P<0.01.
(TIF)

Figure S5 **Established MTG8 and MTG16 colorectal cancer mutations do not alter repression of Kaiso target promoters.** A. 4×KBS artificial promoter assays in HCT116 cells. The graph shows the fold-change in luciferase activity relative to the standard pGL4-TK hRLUC after transfection of expression plasmids encoding the indicated MTG8 mutant constructs. B. HMAT-2.3 artificial promoter assays in HCT116 cells. The graph shows the fold-change in luciferase activity relative to the standard pGL4-TK hRLUC after transfection of expression plasmids encoding the indicated MTG8 mutant constructs. The error bars represent the standard error of three replicate experiments performed in triplicate. *P<0.05, **P<0.01.
(TIF)

Figure S6 **HT29 cells express higher levels of** ***MMP-7*** **and lower levels of** ***MTG16*** **than other colon cancer cell lines.** A. *MTG16*, B. *MMP-7*, and C. *Kaiso* mRNA expression in colon cancer cell lines. The graph shows the fold-change (ΔΔCt) of mRNA. Error bars represent the standard error for three replicate experiments performed in triplicate.
(TIF)

Figure S7 **MTG family members repress endogenous** ***MMP-7*** **expression in Cos7 cells.** *MMP-7* mRNA expression after transfection of the indicated MTG family member in Cos7 cells. The graph shows the fold-change (^ΔΔ^Ct) of *MMP-7* mRNA compared to an empty control. Error bars represent the standard error for three replicate experiments performed in triplicate. ***P<0.001.
(TIF)

Text S1 **Supporting** **Materials and Methods** **.**
(DOCX)
