# Supplementary figures and images for: Kaiso Directs the Transcriptional Corepressor MTG16 to the Kaiso Binding Site in Target Promoters
Source: PLoS One. 2012 Dec 12;7(12):e51205. doi: 10.1371/journal.pone.0051205 (PMC3521008; doi:10.1371/journal.pone.0051205)

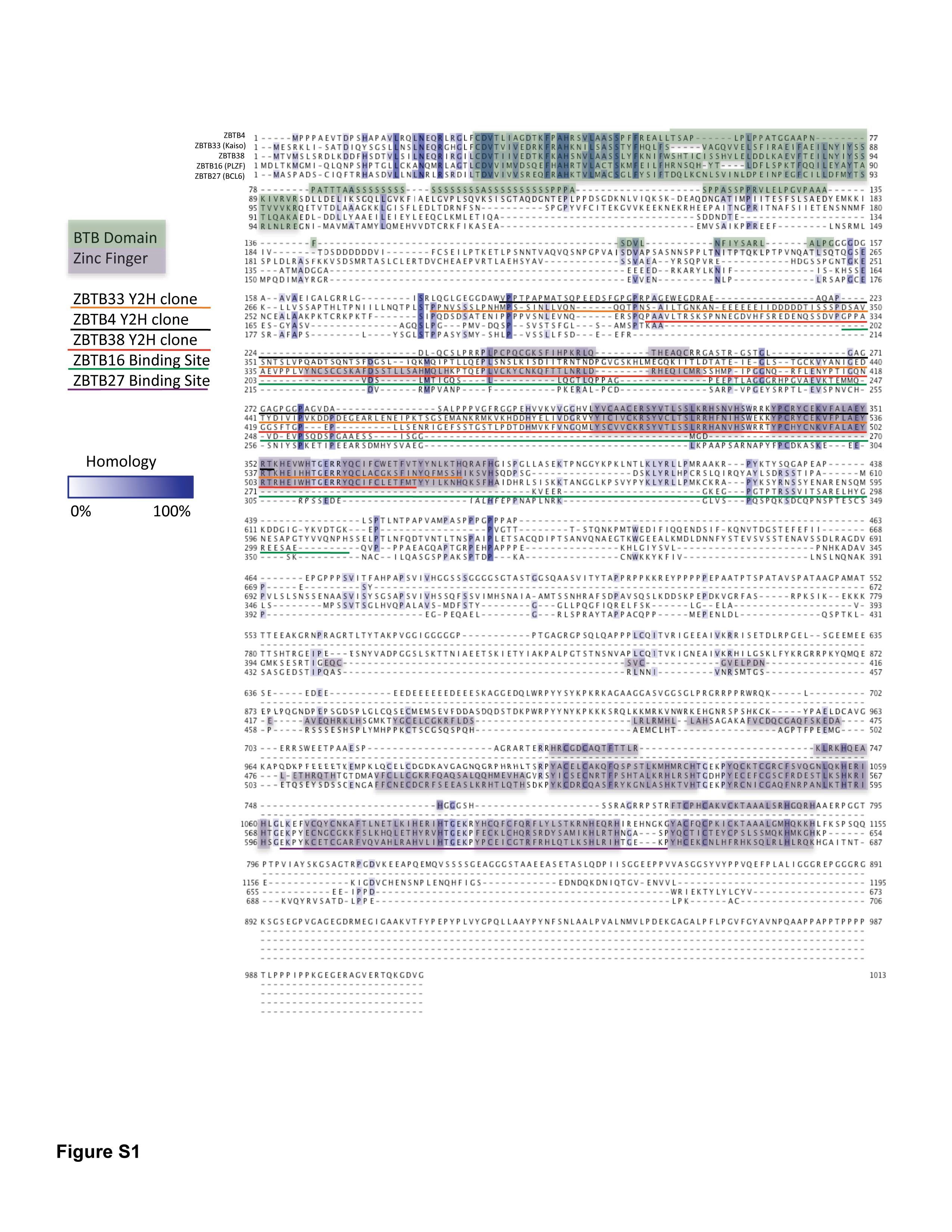

Supplement: Figure S1 — ZBTB family members interact with Kaiso through their homologous zinc finger domains. Uniprot alignment between the five ZBTB family members known to bind MTG16 indicates homology within the zinc fingers (highlighted in purple) and BTB (highlighted in green) domains of each protein (white indicates 0% homology and dark blue indicates 100% homology). Yeast-two hybrid assay indicates binding of Kaiso (orange underline) ZBTB4 (black underline) and ZBTB38 (red underline) to MTG16 within the homologous zinc finger region in a similar manner to ZBTB16 (green underline). (TIF) [file pone.0051205.s001.tif]

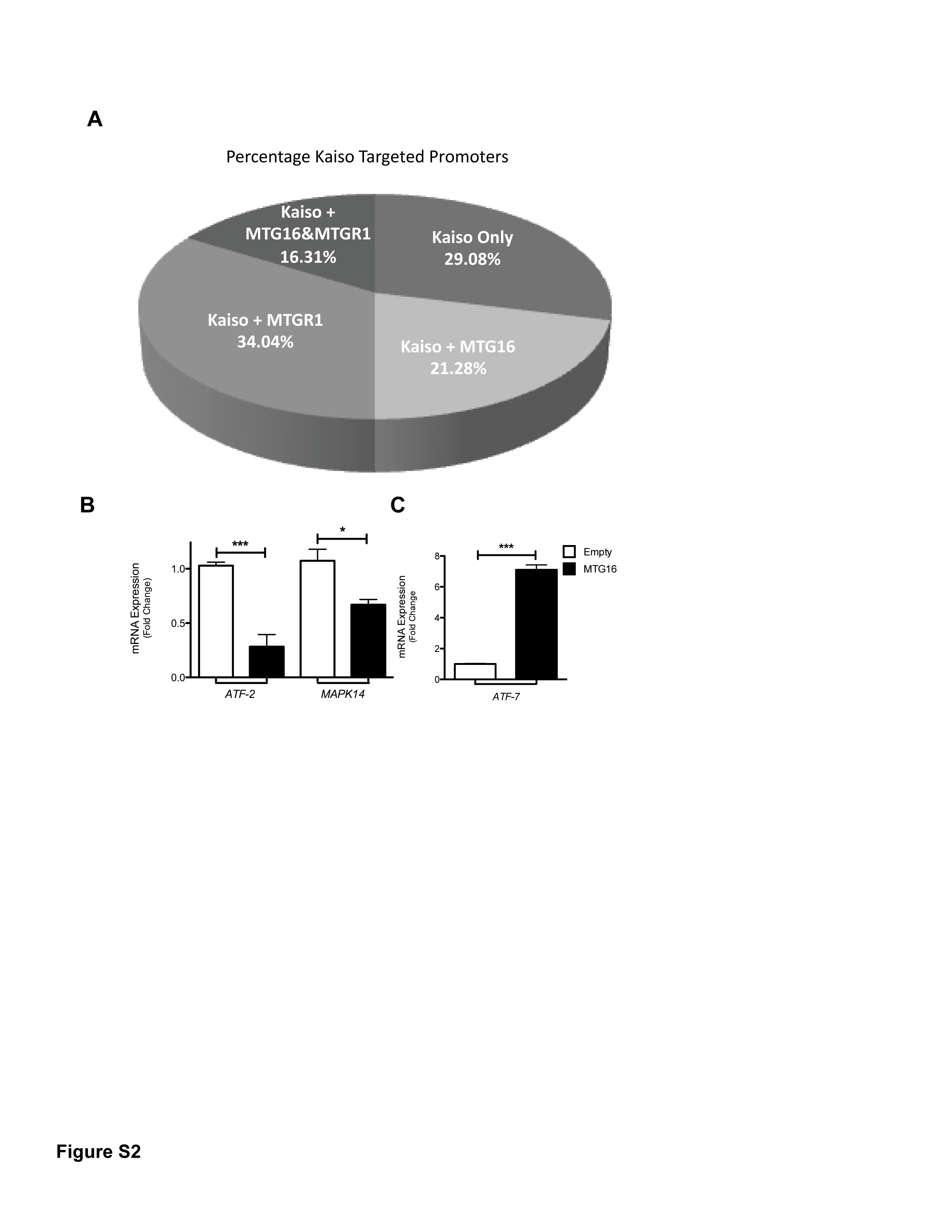

Supplement: Figure S2 — Kaiso shares repression targets with MTG16 and MTGR1. A. The UCSC genome browser ChIP-seq data was used to identify Kaiso targets. Chip-seq data developed by Soler et al., 2011 [26] was used to identify MTG16 and MTGR1 targets that overlapped with Kaiso binding sites. B. ATF-2, MAPK14, and C. ATF-7 mRNA expression upon overexpression of MTG16. The graph shows the fold-change (ΔΔCt) of mRNA compared to an empty vector control. Error bars represent the standard error for three replicate experiments performed in triplicate. *P<0.05, ***P<0.001. (TIF) [file pone.0051205.s002.tif]

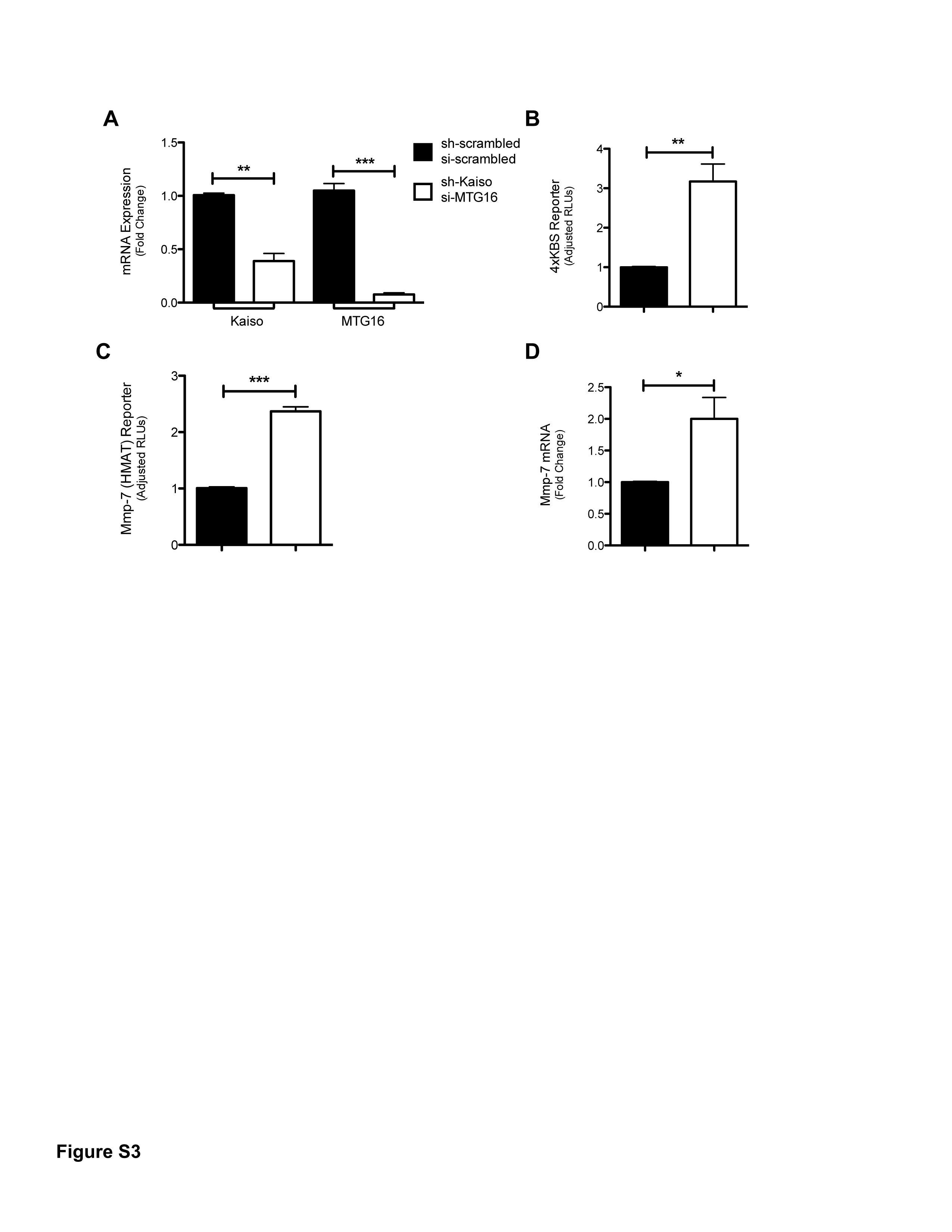

Supplement: Figure S3 — Repression of 4×KBS, HMAT, and MMP-7 is decreased with knockdown of MTG16 and Kaiso in HCT116 cells. A. Kaiso or MTG16 mRNA expression after knockdown of Kaiso (sh-Kaiso) and MTG16 (si-MTG16) in HCT116 cells. The graph shows the fold-change (ΔΔCt) of mRNA compared to a scrambled control (sh-scrambled and si-scrambled). B. 4×KBS and C. HMAT reporter activity after knockdown of both Kaiso and MTG16. D. MMP-7 expression in response to knockdown of Kaiso and MTG16. Error bars represent the standard error for three replicate experiments performed in triplicate. **P<0.01, ***P<0.001. (TIF) [file pone.0051205.s003.tif]

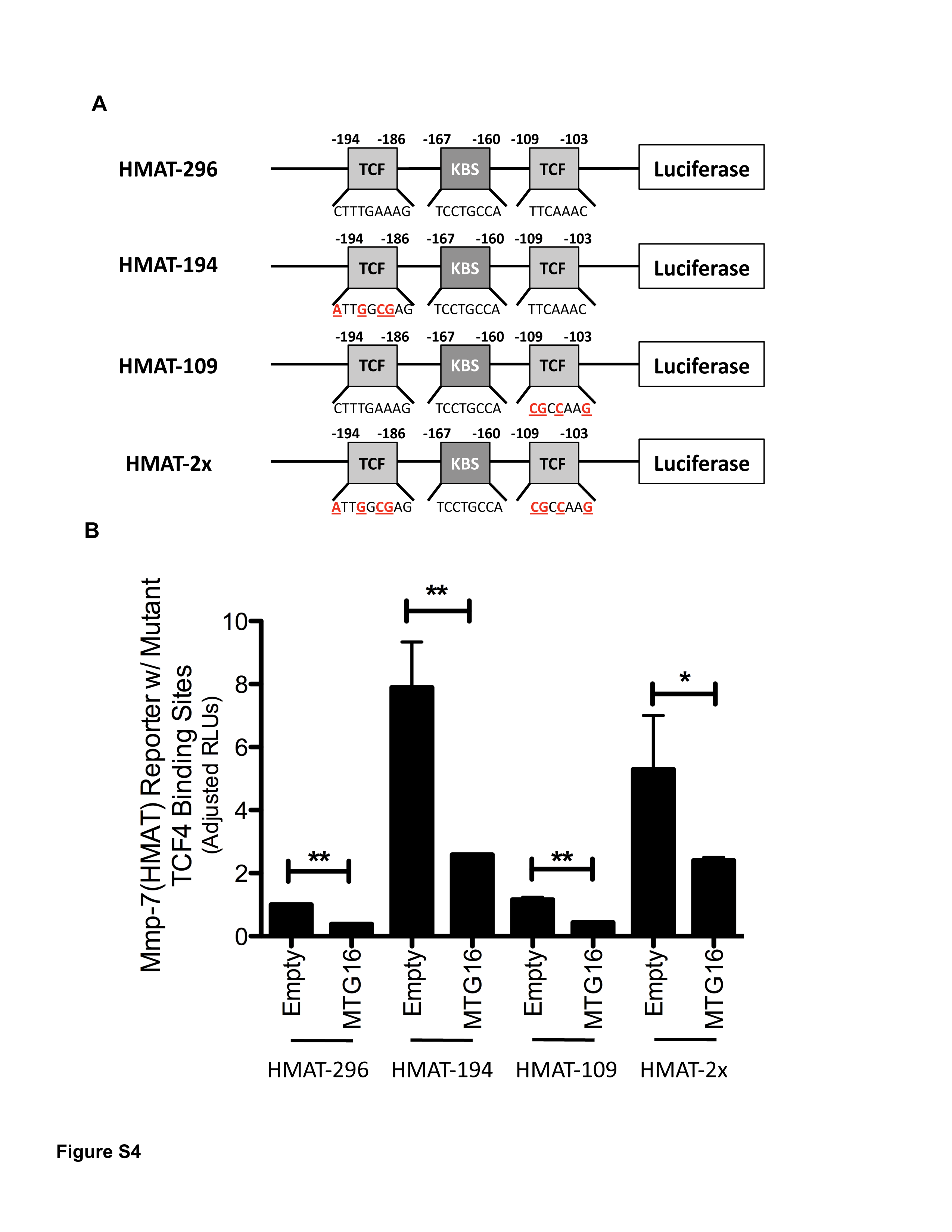

Supplement: Figure S4 — MTG16 does not repress the MMP-7 reporter via TCF binding sites. A. The composition of the truncated MMP-7 reporter (HMAT-296) and TCF mutant reporters (HMAT-194, HMAT-109, and HMAT-2×). B. HMAT artificial promoter assays in HCT116 cells. HCT116 cells were transfected with either 500 ng of MTG16 or 500 ng of Empty vector as a control. The graph shows the fold-change in luciferase activity relative to the standard pGL4-TK hRLUC after transfection of expression plasmids. The error bars represent the standard error of four replicate experiments performed in triplicate. *P<0.05, **P<0.01. (TIF) [file pone.0051205.s004.tif]

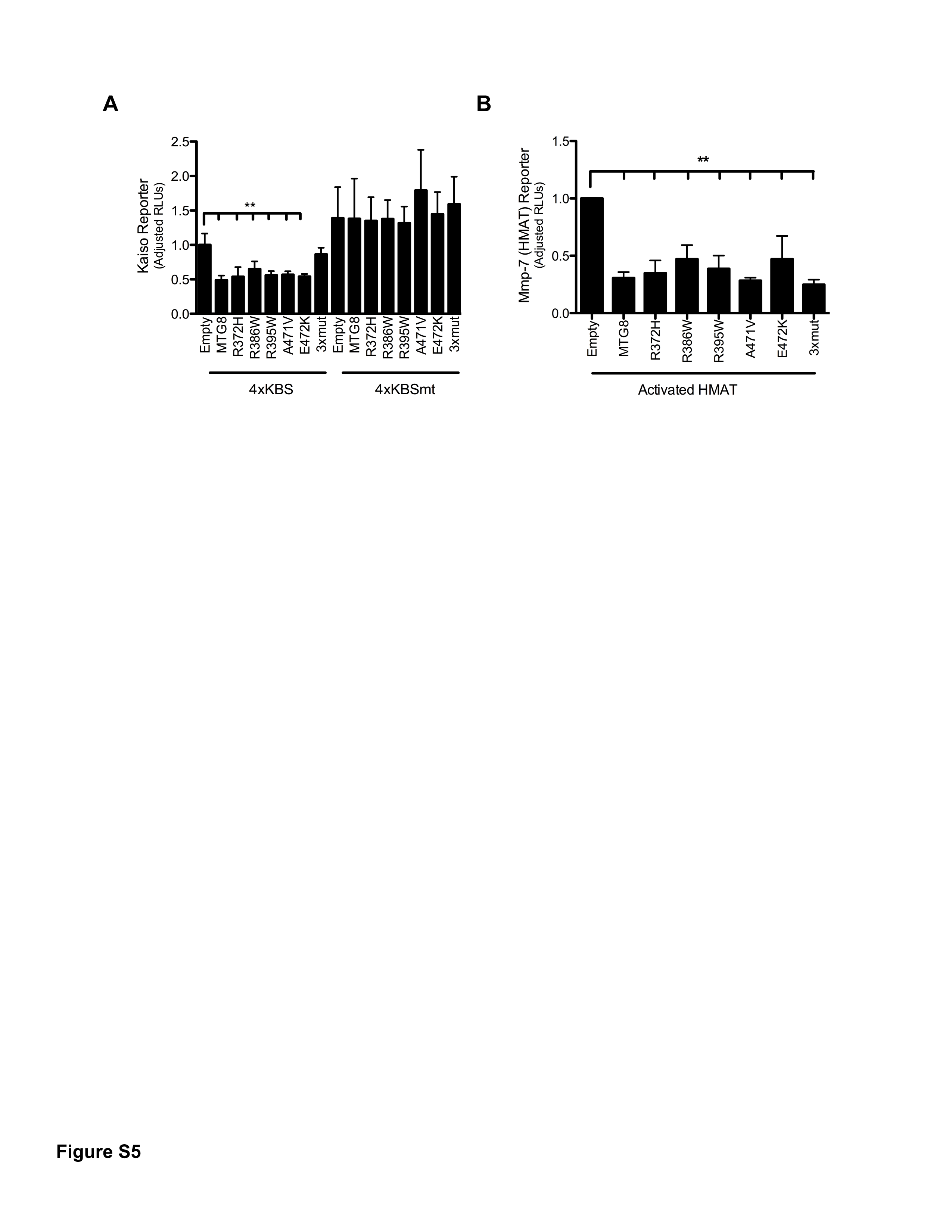

Supplement: Figure S5 — Established MTG8 and MTG16 colorectal cancer mutations do not alter repression of Kaiso target promoters. A. 4×KBS artificial promoter assays in HCT116 cells. The graph shows the fold-change in luciferase activity relative to the standard pGL4-TK hRLUC after transfection of expression plasmids encoding the indicated MTG8 mutant constructs. B. HMAT-2.3 artificial promoter assays in HCT116 cells. The graph shows the fold-change in luciferase activity relative to the standard pGL4-TK hRLUC after transfection of expression plasmids encoding the indicated MTG8 mutant constructs. The error bars represent the standard error of three replicate experiments performed in triplicate. *P<0.05, **P<0.01. (TIF) [file pone.0051205.s005.tif]

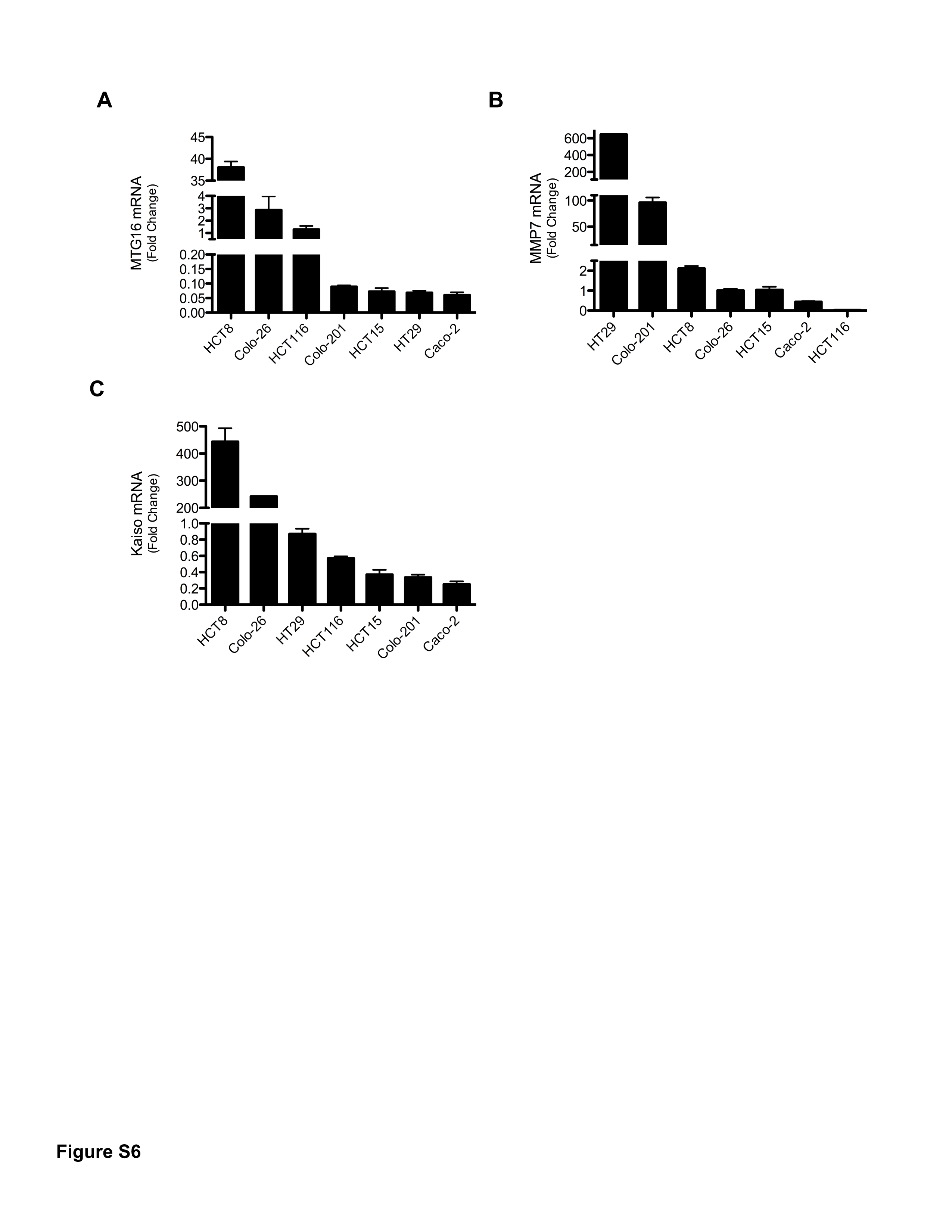

Supplement: Figure S6 — HT29 cells express higher levels of MMP-7 and lower levels of MTG16 than other colon cancer cell lines. A. MTG16, B. MMP-7, and C. Kaiso mRNA expression in colon cancer cell lines. The graph shows the fold-change (ΔΔCt) of mRNA. Error bars represent the standard error for three replicate experiments performed in triplicate. (TIF) [file pone.0051205.s006.tif]

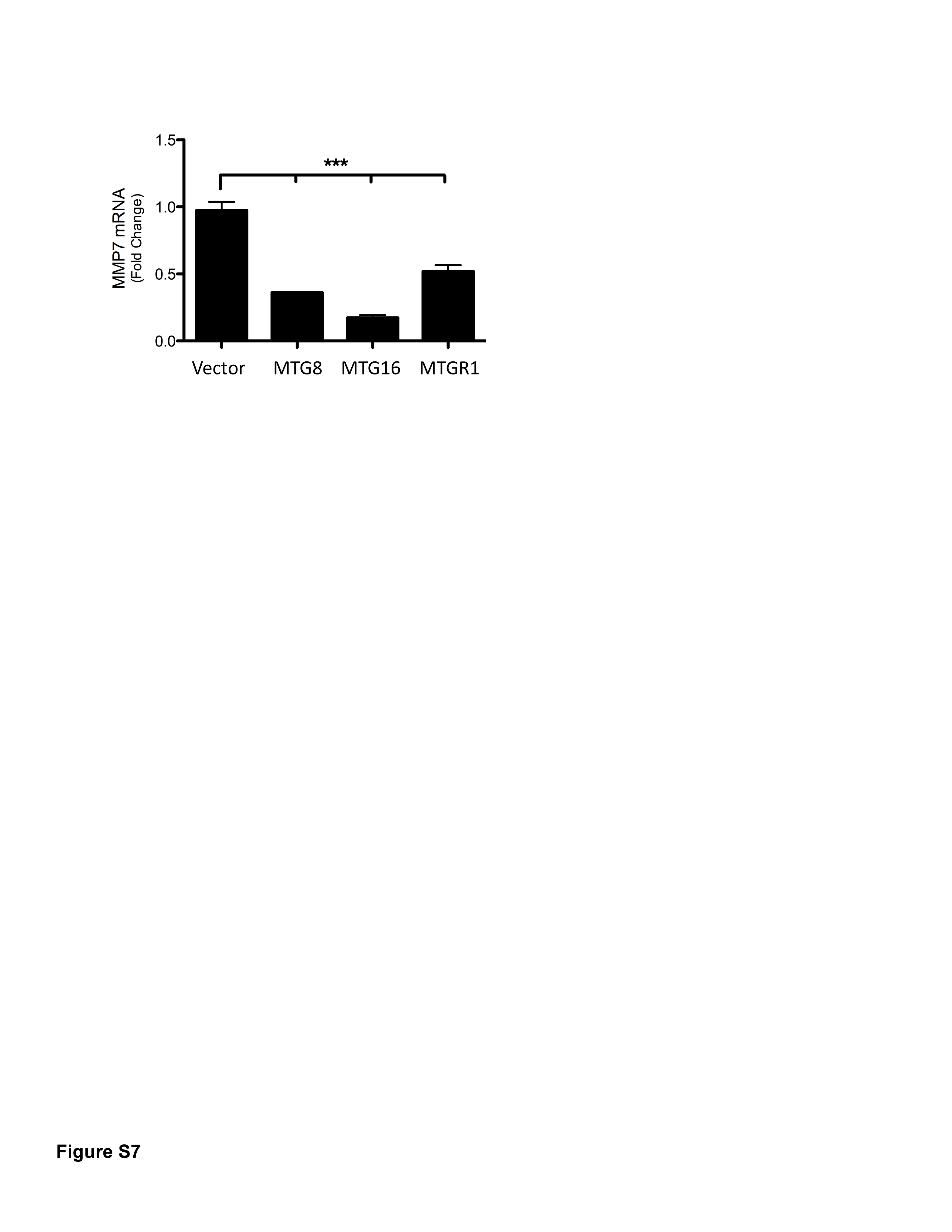

Supplement: Figure S7 — MTG family members repress endogenous MMP-7 expression in Cos7 cells. MMP-7 mRNA expression after transfection of the indicated MTG family member in Cos7 cells. The graph shows the fold-change (ΔΔCt) of MMP-7 mRNA compared to an empty control. Error bars represent the standard error for three replicate experiments performed in triplicate. ***P<0.001. (TIF) [file pone.0051205.s007.tif]
