# Supplementary material for: Kaiso Directs the Transcriptional Corepressor MTG16 to the Kaiso Binding Site in Target Promoters
Source: PLoS One. 2012 Dec 12;7(12):e51205. doi: 10.1371/journal.pone.0051205 (PMC3521008; doi:10.1371/journal.pone.0051205)
Supplement: Text S1 — Supporting Materials and Methods . (DOCX) [file pone.0051205.s008.docx]

**Supplementary Materials and Methods**

*ZBTB Family Alignment*

ZBTBs 4, 16, 27, 33, and 38 (Uniprot identifiers Q9P1Z0, Q05516, P41182, Q86T24, and Q8NAP3, respectively) were aligned using the Uniprot Align program (<http://www.uniprot.org/align>). Alignments were processed using Jalview to identify regions of homology.

*Kaiso and MTG Target Identification*

Kaiso targets were identified using the UCSC Genome Browser Track Search feature. Genes were identified as Kaiso targets if Kaiso bound to the promoter region of the gene (within 1,000 bases of the ATG start site). Highly homologous regions within the identified Kaiso binding sites were established and analyzed for MTG16 or MTGR1 binding using IGV 2.0 and previously established data (21). If MTG binding occurred within the region highlighted as a Kaiso binding site, the promoter was identified as an overlapping target.

*Kaiso and MTG16 Target RT-PCR*

*ATF-2* primers (Fwd: 5’-ACACCCCCATTTATTAAAACACC-3’ and Rev: 5’- ACACCCCCATTTATTAAAACACC-3’) derived from Arora et al. 2011. MAPK14 primers were purchased from RealtimePrimers.com (catalog number VHPS-5546). MTG16 was overexpressed in HT29 cells as performed in the *MMP-7* analysis and RNA was isolated using the Qiagen RNA Isolation Kit and cDNA was generated from 1 μg of total RNA using the iScript cDNA Synthesis Kit (BioRad). RT-PCR was performed using Sybr Green (Invitrogen) reactions according to manufacturer recommendations, and expression was normalized to *GAPDH*. Analysis was performed using the delta-delta Ct method.

*Kaiso and MTG16 Knockdown*

Kaiso knockdown cell lines (see Methods section) were transfected with either MTG16-specific or scrambled siRNA (Dharmacon, L-017195-00 and D-001810-01, respectively) using RNAiMax (Invitrogen) according to manufacturer specifications. Cells were then either collected for RNA analysis or transfected with 4xKBS or HMAT and control reporters (see Methods).
